# Supplementary material for: Spatial and Temporal Variability of Saxitoxin-Producing Cyanobacteria in U.S. Urban Lakes
Source: Toxins (Basel). 2024 Feb 1;16(2):70. doi: 10.3390/toxins16020070 (PMC10892283; doi:10.3390/toxins16020070)
Supplement: Supplementary file 1 [file toxins-16-00070-s001.zip › toxins-2819276-supplementary.pdf]

Supplementary Materials: Spatial and Temporal Variability of Saxitoxin-Producing Cyanobacteria in U.S. Urban Lakes

Table S1. Sampling sites information.

		**Latitude**	Longitude
**Cincinnati, OH, USA**	**Campbell Lake**	**39.23564**	-84.7956
	Winton Woods Lake	39.25898	-84.4951
	Parky's Farm	39.25135	-84.533
	Sharon Woods Lake	39.28554	-84.3891
Kansas City, MO, USA	South Lake	38.97211	-94.6734
	Tomahawk Creek Lake	38.92319	-94.626
	Lake of the Woods	38.99513	-94.5194
	Chaumiere Lake	38.17292	-94.5385
	Big 11 Lake	38.11745	-94.6374
Denver, CO, USA	Sloans Lake	39.75176	-105.048
	Rocky Mt. Lake	39.78239	-105.028

Table S2. Parameters for SYBR® Green qPCR assays.

**Assay**	**Primer sequences (5' to 3')** [a]	**Target**	**T_m (°C)**	**Amplicon (bp)**	**Reference**
Partial gene sequencing	CGCTATACCCACGGATTTGTT<br>GGGATCAGCAGTAGTCCATCTA	*sxtA*	60	395	(Lu et al., 2019)
qPCR	GCGGGACTTTATGCTCTACTAC<br>TACTCCGTCATCGGCATTTG	*sxtA*	60	111	This study

[a]: For each assay, the sense primer is listed in the first line, while the anti-sense primer is listed in the second line.
T_m: Annealing temperature of the qPCR assay. .

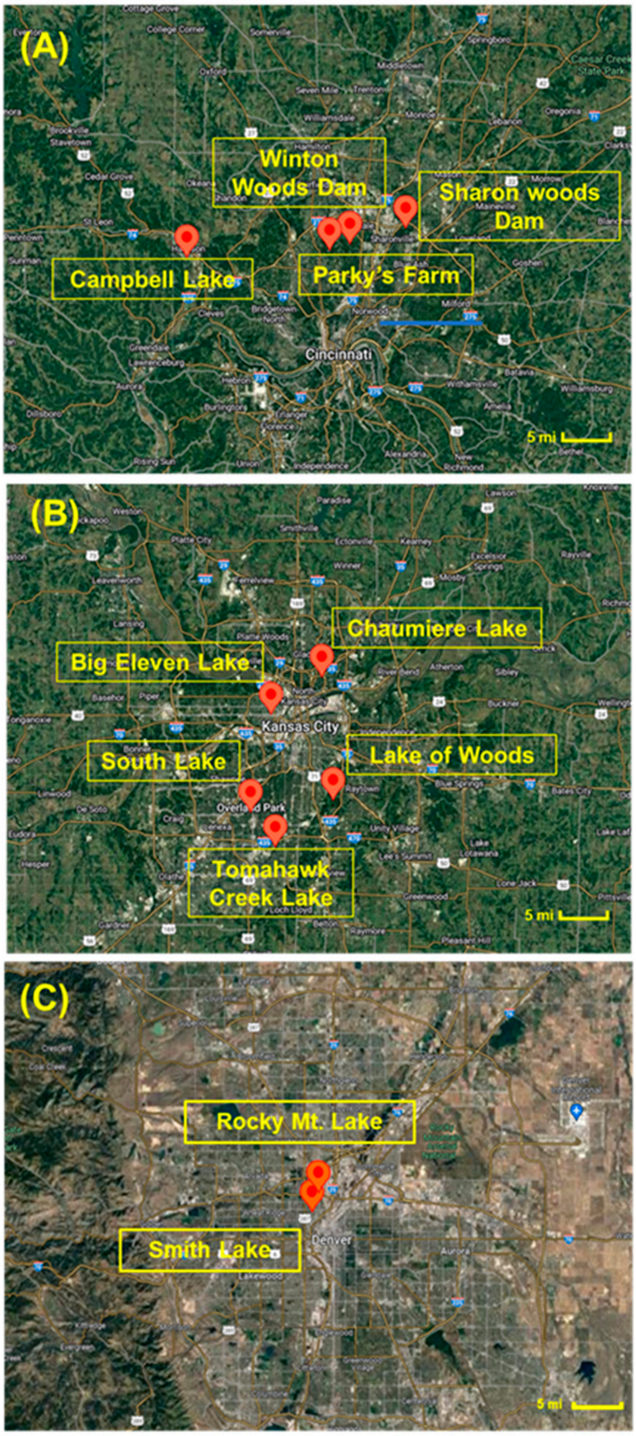

Figure S1. Sampling sites in the urban lakes of Cincinnati (A), Kansas City (B), and Denver (C).

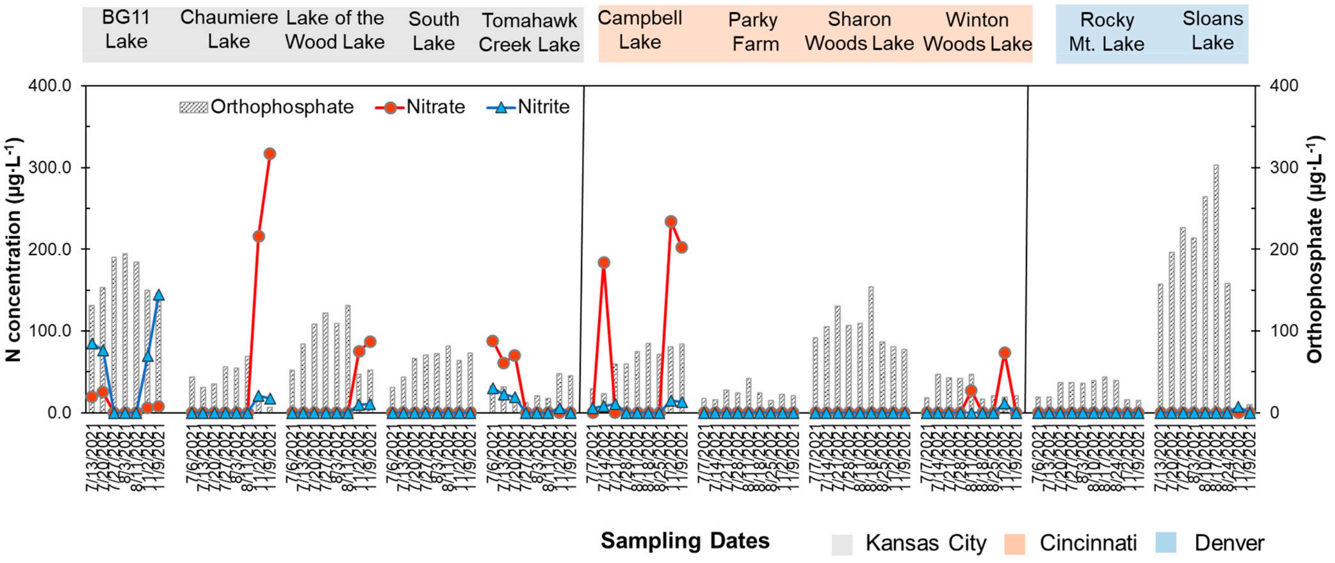

Figure S2. Nitrite (blue), nitrate (red), orthophosphate (gray), and concentrations in the lakes of Kansas City, Cincinnati, and Denver.

Table S3. Table S3. Physicochemical water parameters in the lakes of Kansas City, Cincinnati, and Denver. The numbers below are the average values of the parameters collected from the sampling period, presented as average ± standard deviation.

	Temp. (°C)	Conductivity (uS/cm)	pH	DO (mg/L)	PC(ppb)	CHL (ppb)	PC:CHL	Cell (#/mL)	Alkalinity (mg/L)	Chloride (mg/L)	Sulfate (mg/L)	Nitrate (μg/L)	Nitrite (μg/L)	Orthophosphate as P (μg/L)
Big 11	28.2±1.2	1159±23.3	7.6±0.1	6.14±2.2	127.5±86.1	38.1±18.2	3.2±0.8	5.2E+05	141.8±2.3	193.2±6.97	140±7.61	8.9±12.3	32.1±44.0	170.±27.3
Chaumiere	27.1±0.9	442.3±20.2	8.0±0.2	7.7±1.5	26.4±8.9	59.7±12.4	0.4±0.1	9.8E+04	96.3±3.2	54.6±1.89	30.8±3.06	36.5±40.9	11.9±13.6	21.8±8.3
Lake of the Woods	27.3±1.4	555.3±30.2	7.85±0.2	7.3±3.7	17.8±3.56	90.4±25.8	0.20±0.0	1.3E+04	176.3±6.1	46.1±3.73	28.7±2.14	0±0	0±0	100.9±28.9
South Lake	26.5±1.2	383.8±17.1	7.7±0.2	7.0±5.0	105.9±54.7	48.7±20.5	2.3±1.0	4.5E+05	98.0±7.4	55.7±13.9	12.4±1.26	0±0	0±0	60.7±19.2
Tomahawk Creek Lake	27.4±1.0	350.5±27.2	8.4±0.4	9.8±2.8	90.6±74.0	47.2±17.9	1.94±1.5	2.3E+04	100.4±13.	30.6±5.50	21.1±4.96	0±0	0±0	48.2±14.1
Campbell Lake	24.2±0.9	692±8.7	9.19±0.1	7.6±1.0	83.6±62.4	38.0±13.1	2.13±1.0	5.8E+03	118.1±2.3	103.8±2.54	80.2±1.72	0±0	0±0	216.8±53.5
Parky's Farm	26.6±0.6	869.1±33.6	8.7±0.3	12.0±2.7	13.6±4.2	38.8±23.4	0.4±0.3	3.6E+04	83.5±12.	128±6.02	150.±7.20	526±96	29.9±9.31	24.3±10.8
Sharon Woods	25.3±1.4	399.3±22.7	8.9±0.1	10.0±1.4	7.1±9.7	13.4±15.5	0.4±0.3	7.6E+03	100.7±5.7	50.0±5.00	24.8±1.83	0±0	0±0	33.6±9.2
Winton Woods	28.7±1.2	278.0±57.0	8.6±0.3	9.9±1.8	25.3±10.0	43.5±11.5	0.5±0.1	7.9E+03	117.0±18.	34.9±16.5	11.1±2.00	3.04±9.13	0±0	31.2±13.1
Rocky Mt. Lake	25.6±2.4	439.2±30.5	8.1±0.0	11.8±2.5	17.7±10.1	37.1±15.1	0.4±0.1	4.4E+03	203.4±18.	140.4±4.16	129.3±3.14	81.5±156.	5.68±7.04	49.7±22.8
Sloans Lake	26.4±0.9	173.9±33.2	7.7±0.4	5.6±1.9	23.4±28.7	40.4±14.6	0.8±1.2	1.1E+05	64.1±6.2	27.9±12.0	0±0	0±0	0±0	22.7±8.9

References

Lu, J.; Zhu, B.; Struewing, I.; Xu, N.; Duan, S. Nitrogen–phosphorus-associated metabolic activities during the development of a cyanobacterial bloom revealed by metatranscriptomics. Sci. Rep. 2019, 9, 2480.
